# Episignature leads to diagnosis and reclassification of DYRK1A variant in a child with syndromic neurodevelopmental disorder: a case report

**DOI:** 10.3389/fgene.2026.1813300

**Published:** 2026-05-22

**Authors:** Inas Al-Younis, Laurence Basque, Nicolas Crapoulet, Sarah Dyack, Francisco del Caño-Ochoa, Santiago Ramón-Maiques, Sara B. MacKay, Jessica Rzasa, Bekim Sadikovic, Haley McConkey, Mouna Ben Amor

**Affiliations:** 1 Vitalité Health Network, Dr Georges-L.-Dumont University Hospital Center, Moncton, NB, Canada; 2 Department of Chemistry and Biochemistry, New Brunswick Centre for Precision Medicine, Université de Moncton, Moncton, NB, Canada; 3 Centre de Formation Médicale du Nouveau Brunswick, Moncton, NB, Canada; 4 Division of Medical Genetics, Department of Pediatrics, Dalhousie University, Halifax, NS, Canada; 5 Instituto de Biomedicina de Valencia (IBV), Consejo Superior de Investigaciones Científicas (CSIC), Valencia, Spain; 6 Centro de Investigacion Biomédica en Red de Enfermedades Raras (CIBERER)–Instituto de Salud Carlos III, Valencia, Spain; 7 Valencia Biomedical Research Foundation, Centro de Investigación Príncipe Felipe (CIPF) - Associated Unit to the Instituto de Biomedicina de Valencia (IBV), Valencia, Spain; 8 Maritime Medical Genetics Service, Izaak Walton Killam (IWK) Health Centre, Halifax, NS, Canada; 9 Verspeeten Clinical Genome Centre, London Health Sciences Centre, London, ON, Canada; 10 Department of Pathology and Laboratory Medicine, Western University, London, ON, Canada

**Keywords:** case report, diagnostic odyssey, DNA methylation, DYRK1A, episignatures, neurodevelopmental disorder, variant of uncertain significance (VUS), whole-genome sequencing

## Abstract

**Background:**

Neurodevelopmental disorders (NDDs) are genetically heterogeneous, and standard genomic testing frequently yields variants of uncertain significance (VUS) or misses cryptic structural variants. Episignature analysis, which detects disorder specific genome-wide DNA methylation patterns, has emerged as a functional tool to resolve diagnostic uncertainty.

**Case presentation:**

We report a 7-year-old female with global developmental delay, autism spectrum disorder, epilepsy, microcephaly, and a distinctive facial gestalt. Initial trio whole-exome sequencing identified inconclusive VUSs in *CAD* and *POLR1A*. A therapeutic trial of uridine for the *CAD*-related variants provided only transient benefit. Genome-wide DNA methylation analysis via EpiSign revealed a positive result for the episignature for Intellectual Developmental Disorder, Autosomal Dominant 7 (MRD7), associated with DYRK1A haploinsufficiency. Guided by this result, trio whole-genome sequencing identified a *de novo* heterozygous deletion involving exon 5 of *DYRK1A*. The exact breakpoints were chr21:38,852,074–38,856,009 (GRCh37/hg19) and chr21:39,044,532–39,048,467 (GRCh38/hg38). This variant was reclassified from VUS to likely pathogenic based on concordant episignatures findings, clinical phenotype, and *de novo* occurrence.

**Conclusion:**

This case highlights the utility of episignature analysis to help diagnose NDD patients, guide further molecular diagnostic testing, and as a functional assay to aid variant reclassification. Integration of epigenomic profiling with standard of care testing can shorten the diagnostic odyssey, prevent unnecessary interventions, and directly inform clinical management in patients with unresolved NDDs.

## 1 Introduction

Neurodevelopmental disorders (NDDs) encompass a diverse array of conditions characterized by cognitive, behavioral, linguistic, and motor deficits. They may also present with epilepsy and other psychosocial/pediatric syndromic comorbidities. Their etiology is often genetic, and referrals for genetic evaluation and testing are appropriate when developmental delays or regressions are noted early in life. The phenotypic overlap among different NDDs, however, poses a major diagnostic challenge, often resulting in a protracted diagnostic odyssey for affected families (Wright et al., 2018; Pandey et al., 2025).

Chromosomal microarray and exome sequencing have revolutionized the diagnostic landscape for NDDs, increasing diagnostic yield to over 30% in some cohorts (Pandey et al., 2025; Vissers et al., 2017). Yet these technologies still do not capture all potentially causative variants, such as small deletions in exons and deep intronic mutations. In addition, many genetic findings are classified as Variants of Uncertain Significance (VUS), leaving clinicians without a clear path for interpretation and clinical management.

As a strategy to overcome these diagnostic limitations, genome-wide DNA methylation analysis has emerged as a powerful tool in clinical genetics, particularly through the development of disease-specific DNA methylation patterns, called “episignatures.” These episignatures can functionally validate the pathogenicity of genomic variants, help prioritize candidate genes, and, in certain cases, reveal diagnoses that conventional sequencing alone may miss (Aref-Eshghi et al., 2020; Sadikovic et al., 2021). EpiSign is a validated genome-wide DNA methylation-based assay that detects disorder-specific episignatures across a broad range of conditions (more than 125 episignatures assessed on EpiSign version 5), particularly those with a neurodevelopmental presentation (McConkey and Sadikovic, 2024). EpiSign analyzes genome-wide DNA methylation in peripheral blood by comparing a patient’s methylation changes to thousands of reference cases in the EpiSign Knowledge database (EKD) to determine if the patient’s DNA methylation pattern matches a known episignature. These episignatures serve as highly sensitive and specific biomarkers for various rare diseases, including NDDs. The utility of EpiSign was recently demonstrated in a large international study involving 2,399 cases analyzed between May 2019 and January 2023. The study found a 18.7% positive detection rate for cases undergoing a comprehensive screen and a 32.4% positivity rate in those who had a targeted assessment for a specific suspected disorder (Kerkhof et al., 2024). These findings underscore the significant potential of episignature analysis as a complementary diagnostic method for patients with suspected NDDs. Use of DNA methylation analysis is especially relevant in extremely rare or newly defined disorders, where phenotypic expression may be variable or evolve over time.

One such condition is caused by pathogenic variants in the *DYRK1A* (dual-specificity tyrosine-phosphorylation-regulated kinase 1A), located within the Down syndrome critical region on chromosome 21q22.13. The DYRK1A protein plays an essential role in neural progenitor proliferation, synaptogenesis, and brain size regulation (Dang et al., 2018; Feki and Hibaoui, 2018). *DYRK1A* haploinsufficiency is responsible for Intellectual developmental disorder, autosomal dominant 7 (MRD7, OMIM #614104). Intellectual developmental disorder, autosomal dominant 7 is a recognizable clinical syndrome defined by microcephaly, global developmental delay, profound speech delay, epilepsy, feeding difficulties, and characteristic facial morphology (Bronicki et al., 2015; Ji et al., 2015). While most pathogenic *DYRK1A* variants are truncating and *de novo*, subtle structural variants such as small exon deletions may evade detection by exome sequencing. An episignature has been developed for Intellectual developmental disorder, autosomal dominant 7 (Kerkhof et al., 2024), which may be helpful in identifying such cases (Ji et al., 2015).

Herein, we present the case of a female proband with neurodevelopmental delay, autism spectrum disorder, epilepsy and microcephaly, in whom exome sequencing revealed inconclusive VUS findings in the *CAD* and *POLR1A*. Limited benefit was obtained from uridine therapy and these variants were reclassified as likely benign with functional testing. In parallel, the patient was enrolled in the Canadian national Episign-CAN study where EpiSign analysis was performed and identified an Intellectual developmental disorder, autosomal dominant 7 episignature. Thereafter, trio whole-genome sequencing was performed, and a *de novo* heterozygous deletion of *DYRK1A* exon 5 was detected. This variant, initially a VUS, was subsequently reclassified as likely pathogenic. These epigenetic findings helped the clinical genetics team correlate a subtle and not easily recognisable phenotype to an established condition. This case highlights how the combination of epigenomics and advanced sequencing can help improve clinical certainty in NDD patients with ambiguous clinical presentations.

## 2 Case presentation

We report a female child with neurodevelopmental delay in whom episignature analysis allowed for a prompter diagnosis and guided the reclassification of a *DYRK1A* variant.

### 2.1 Personal and family history

The proband was a 7-year-old girl who was referred for genetic evaluation of a neurodevelopmental disorder. She was first assessed by genetics at 3.5 years of age. She was the product of a spontaneous conception and an uncomplicated pregnancy, delivered vaginally at 41 weeks with a birth weight of 3.2 kg. Her clinical history included autism spectrum disorder, which was diagnosed at 18 months, global developmental delay, epilepsy with febrile and absence seizures beginning at 2 years, as well as severe food aversion. She received multidisciplinary care, including physiotherapy, occupational therapy, speech therapy, dietician support, and autism interventions. At initial evaluation, she displayed distinctive facial features including prominent supraorbital ridges, deep set eyes, a high nasal bridge, short philtrum, and mild retrognathia. Growth parameters included height 94 cm (Z = −1.2), weight 13.7 kg (Z = −0.6), and head circumference 45 cm (Z = −2.9), consistent with marked microcephaly. The remainder of the physical exam was unremarkable. Family history was notable for a healthy maternal half-sister, a mother with asthma, and a healthy father with no reported consanguinity. A paternal great-aunt reportedly had developmental delay and epilepsy, and died at age 30 years.

### 2.2 Clinical investigations

Brain MRI demonstrated generalized cerebral atrophy. SNP chromosomal microarray analysis (CMA) did not reveal any copy number variation. Methylation-Specific MLPA (Multiplex Ligation-dependent Probe Amplification) analysis of *SNRPN* did not detect any characteristic methylation changes suggestive of Angelman syndrome. A broad metabolic screen for creatine deficiency disorders, Smith-Lemli-Opitz syndrome, congenital disorders of glycosylation, aminoacidopathies, organic acidurias, lysosomal storage disorders, mucopolysaccharidoses, and purine/pyrimidine metabolism disorders did not identify any significant disturbances.

Phenotype-driven trio whole-exome sequencing (WES) was performed in a clinically accredited molecular laboratory. The analysis was performed using a clinical pipeline consistent with GRCh37/hg19.

**FIGURE 1:**
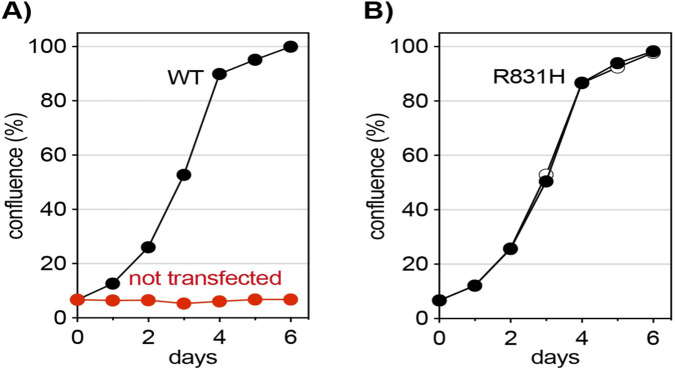
Proliferation of *CAD*-knockout cells in uridine-free medium. Growth complementation assay of *CAD*-KO cells grown without uridine showed that the non-transfected cells failed to proliferate, whereas transfection with GFP-CAD WT **(A)** or the p. R831H variant **(B)** similarly rescued the growth defects of *CAD*-KO cells, indicating that the variant is likely benign. All conditions were assayed in duplicate. All panels represent original data generated in this study.

The WES findings were inconclusive with the identification of three VUSs: one synonymous and one missense in *CAD* (in trans) and one in *POLR1A* (*de novo*). The *POLR1A* variant was not clinically consistent with Cincinnati-type acrofacial dysostosis and was not pursued further. Given that biallelic *CAD* variants can cause congenital disorder of glycosylation type IZ, the family elected to trial oral uridine supplementation (50 mg/kg/day in three divided doses). Transient improvements in attention, vocalization, and sensory tolerance were observed, but these plateaued after few months. The trial was discontinued after 6 months. Functional assays revealed that *CAD* missense variant p. Arg831His restored the development of *CAD*-knockout cell lines in uridine-free media to wild-type levels (Figure 1), supporting its classification as likely benign and ruling out CAD deficiency as the source of the patient’s phenotype.

In the absence of a confirmed diagnosis, the case was enrolled in the EpiSign-CAN national research study to obtain EpiSign analysis. Results of EpiSign analysis prompted whole genome sequencing for diagnostic confirmation.

## 3 Methodology

### 3.1 CAD functional studies

The pathogenicity of the *CAD* variant p. Arg831His was evaluated as described previously (Del Caño-Ochoa et al., 2023; Del Caño-Ochoa et al., 2020). In brief, *CAD-*knockout U2OS cells, which require uridine for growth, were transfected with pcDNA3.1-GFPhuCAD encoding either wild-type protein or the p. Arg831His variant. After confirming *CAD* expression by fluorescent microscopy, cells were cultured in uridine-free media and monitored for proliferation over 1 week. This growth complementation assay identifies mutations that impair CAD function, while variants supporting wild-type-like growth are considered likely benign. Growth-complementation experiments revealed that expression of the *CAD* p. Arg831. This variant restored CAD-knockout cell proliferation in uridine-free media to levels comparable to those of wild-type CAD (Figure 1). This result shows the preserved enzymatic function and supports a benign classification of this variant.

### 3.2 Episign™ DNA methylation analysis

The case was enrolled in the EpiSign-CAN national study, which included 16 Medical Genetics Clinics and Molecular Diagnostics Laboratories across Canada, aimed at assessing the diagnostic and clinical utility of EpiSign analysis. A peripheral blood DNA sample was sent to London Health Sciences Centre Molecular Diagnostics Laboratory, the Lead Site for this study, for data generation and analysis. DNA methylation data was generated using the Illumina Infinium EPIC BeadChip array following previously described methods (Levy et al., 2022). The DNA methylation data were processed using the validated EpiSign (version 5) assay (Sadikovic et al., 2021; Kerkhof et al., 2024), which assesses 126 genes/genetic regions corresponding to 114 conditions.

### 3.3 Trio whole-genome sequencing

Trio whole-genome sequencing (WGS) was performed at CENTOGENE (Rostock, Germany) using Illumina paired-end sequencing at an average coverage of ∼30×. Reads were aligned to GRCh37/hg19 and the human mitochondrial reference genome (rCRS, NC_012920). Variant calling included SNVs and indels using the DRAGEN pipeline (Illumina). Structural variants were detected using Manta, and copy number variants (CNVs) were identified using DRAGEN in combination with in-house algorithms. Additional analyses included screening for repeat expansions, SMA, GBA1 recombinations, and uniparental disomy (UPD). The detailed methods of this WGS analysis, have been included in the Supplementary Material.

## 4 Results of episignatures and confirmatory molecular findings

**FIGURE 2:**
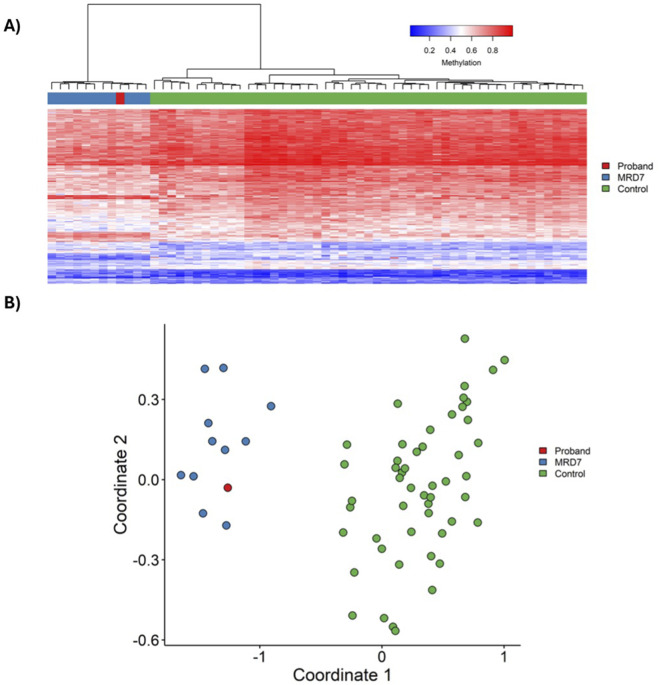
EpiSign DNA methylation analysis of peripheral blood DNA from the proband. **(A)** Euclidian hierarchical clustering and **(B)** multidimensional scaling plots indicate that the case (red) has a DNA methylation episignatures similar to subjects with a confirmed Intellectual developmental disorder, autosomal dominant 7 episignatures (blue) and distinct from controls (green). Each row of the hierarchical clustering heatmap represents one CpG probe on the DNA methylation array that is used in the Intellectual developmental disorder, autosomal dominant 7 episignatures, and each column represents one individual’s sample. The heatmap color scale from blue to red represents the DNA methylation level (beta value) from 0 (no methylation) to 1 (fully methylated).

**FIGURE 3:**
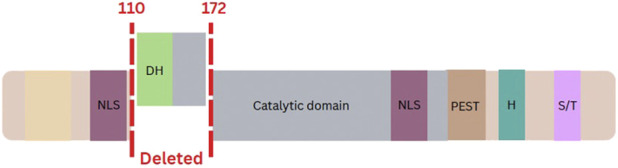
Schematic of the *DYRK1A* exon 5 deletion and functional impact. The identified *de novo* heterozygous deletion of exon 5 (chr21:38,852,055–38,856,028; hg19) is depicted. This deletion disrupts critical protein domains, including the homodimerization and kinase domains, and was functionally validated by a positive result for the Intellectual Developmental Disorder, Autosomal Dominant specific DNA methylation episignature (EpiSign), leading to its reclassification from a variant of uncertain significance (VUS) to likely pathogenic.

Genome-wide DNA methylation analysis by EpiSign, version 5, was performed on peripheral blood. Analysis revealed a methylation pattern strongly consistent with the Intellectual Developmental Disorder, Autosomal Dominant 7 episignature, associated with *DYRK1A* haploinsufficiency. The MVP score is derived from a supervised support vector machine classification model and provides a continuous probability value reflecting concordance with a given episignature. A score of 0.99 indicates strong concordance with the Intellectual Developmental Disorder, Autosomal Dominant 7 episignature. The proband had no other elevated MVP scores for the >120 disorder episignatures assessed by EpiSign version 5. Concordance across all three analytical parameters, MVP score (0.99), Euclidean hierarchical clustering, and multidimensional scaling, constituted a high-confidence positive result for Intellectual Developmental Disorder, Autosomal Dominant 7 (Figure 2) according to published episignature interpretation recommendations (Kerkhof et al., 2024). This high-confidence episignature result directly implicated *DYRK1A* haploinsufficiency and provided a strong rationale to pursue structural variant–sensitive testing. Trio WES was previously performed in a clinically accredited laboratory (Blueprint Genetics, Finland) using a standard clinical pipeline aligned to the GRCh37/hg19 reference genome. Reanalysis of the original WES failed to detect a *DYRK1A* variant, with a low-quality copy number loss affecting this region being flagged initially but not reported, as it was considered likely an artifact due to reduced coverage associated with local sequence characteristics (e.g., low GC content). This underscores the known limitations of WES for reliable detection of single-exon copy number variants. The Episign findings prompted trio WGS, which identified a *de novo* heterozygous deletion of exon 5 in *DYRK1A* (NM_001396.4). Initially classified as a VUS, this *DYRK1A* exon 5 deletion was reclassified as likely pathogenic based on concordant episignature results, and other supportive factors. In fact, within the ACMG/AMP framework (Richards et al., 2015), the episignature provided functional evidence supporting pathogenicity (PS3_supporting), which, when combined with *de novo* occurrence with its phenotype specificity (PS2_supporting to moderate), the allele frequency extremely low in all gnomAD populations (PM2_supporting), prior reports of pathogenic variants in adjacent regions of the gene (Figure 3) and loss-of-function mechanism (PVS1_moderate), supported reclassification to likely pathogenic, therefore establishing a molecular diagnosis of Intellectual Developmental Disorder, Autosomal Dominant 7. This case highlights the diagnostic utility of whole-genome sequencing for detecting variants, particularly single-exon CNVs, that may be missed by exome sequencing.

## 5 Discussion

NDDs impact up to 3% of children globally (Marrus and Hall, 2017) and Mendelian disorders arising from pathogenic changes to a single gene frequently present with overlapping neurodevelopmental features. To further complicate the differential diagnosis for NDDs, frequent new disease gene discoveries add to the list of potential conditions to be assessed and result in the re-evaluation of unsolved patients over time. Furthermore, teratogenic exposures and maternal conditions also contribute to developmental defects and are often considered in the differential diagnosis for NDD patients, further complicating the search for a diagnosis. These challenges, as well as more than 60% of patients left without a diagnosis after standard of care testing (Schwarze et al., 2018; de Ligt et al., 2012; Lemke et al., 2012; Sikkema-Raddatz et al., 2013; Chung et al., 2023; Clark et al., 2018), leads to a prolonged diagnostic odyssey, up to 12 years in Canada (Oei et al., 2017). This negatively impacts patients, families, and the healthcare system. Looking beyond the genetic sequence, to epigenetic changes, presents an opportunity to improve diagnostic yield in these patients.

Intellectual developmental disorder, autosomal dominant 7, presents with NDD phenotypes including intellectual disability, disrupted speech development, autism spectrum disorder, and microcephaly, leading to a dysmorphic facial gestalt, seizures and feeding difficulties (Bregje WM van Bon BPCBB de V and EEE, 2015; Obara et al., 2023; Whitaker and Serrano, 2024). DYRK1A is a highly conserved protein (Bregje WM van Bon BPCBB de V and EEE, 2015) that is known to be important in the proper development of the nervous system, with overexpression being strongly correlated with Down syndrome. Due to the importance of proper dosage of *DYRK1A* during neural development, haploinsufficiency of *DYRK1A* also results in a disruption of important neural processes that lead to the core features of Intellectual Developmental Disorder, Autosomal Dominant, 7 (Dang et al., 2018; Murphy et al., 2024). Reported pathogenic variants in *DYRK1A* are typically result in loss-of-function (Bronicki et al., 2015). Here, the identified *de novo* deletion of exon 5 in our proband impacts the kinase domain of the protein, likely impairing the enzyme’s catalytic activity and disrupting phosphorylation of downstream substrates critical for neuronal differentiation and synaptic maturation (Dang et al., 2018), resulting in the NDD features observed. Although this variant represents an in-frame deletion affecting less than 10% of the protein, it impacts residues within the ATP-binding pocket of the catalytic domain, which is essential for kinase activity. Reported pathogenic missense and truncating variants cluster within the kinase domain, particularly around the ATP-binding site and catalytic core, resulting in impaired or abolished kinase activity and disrupted downstream neuronal developmental pathways (Ji et al., 2015; Courraud et al., 2021). Despite well-documented facial features and phenotype for this condition, it can still be challenging to arrive at a clinical diagnosis, as similar and overlapping features are displayed in other Mendelian neurodevelopmental disorders (Ji et al., 2015; Bregje WM van Bon BPCBB de V and EEE, 2015).

DNA methylation, a dynamic epigenetic modification, is vital for cellular functions and development (McConkey and Sadikovic, 2024; Gr et al., 2019; Reichard and Zimmer-Bensch, 2021; Moore et al., 2013). Research studies have demonstrated that many rare NDD disorders exhibit unique and specific genome-wide DNA methylation patterns, called episignatures (Aref-Eshghi et al., 2020; Sadikovic et al., 2021; Kerkhof et al., 2024; Levy et al., 2022; Schenkel et al., 2018; Aref-Eshghi et al., 2018a; Jones et al., 2013; Aref-Eshghi et al., 2017; Aref-Eshghi et al., 2018b; Cappuccio et al., 2020; Aref-Eshghi et al., 2019; Man et al., 2025; Marshall et al., 2024). EpiSign was originally developed to identify and classify rare Mendelian disorders, particularly genetic NDDs, but has since expanded to include teratogenic exposures and recurrent congenital malformation syndromes (Haghshenas et al., 2024a; Haghshenas et al., 2024b). While EpiSign is considered research use only software in Canada, it is used as a laboratory-developed test internationally, with 9 laboratories now using EpiSign in the diagnostic assessment for patients with suspected genetic rare disease (Kerkhof et al., 2024). This case illustrates an extended diagnostic odyssey, as exemplified by the proband’s extensive clinical evaluation and genetic testing, which ultimately yielded no diagnosis. EpiSign was able to assess for 114 conditions with the hope of finding a new direction for diagnostic testing, which was identified through a strong match to the Intellectual developmental disorder, autosomal dominant 7 episignature. The patient’ss phenotype was a clinical fit, which then allowed the existing exome data to be reinvestigated. When no variant was identified, trio WGS uncovered a structural variant that resulted in the loss of exon 5, which encodes a portion of the protein’s kinase domain (Thompson et al., 2015).

This identified variant was originally considered a VUS; however, supportive functional evidence from the episignature findings, as well as concordant phenotype and literature evidence regarding variant impact, resulted in VUS reclassification. Under the same variant interpretation guidelines, episignatures can also be considered phenotypic (PP4) evidence, which would have led to this variant being recalssifed as pathogenic. In our case, episignature analysis played a pivotal role in linking the exon 5 deletion to the patient’s phenotype by providing the interpreting molecular diagnostics laboratory functional evidence of disease-specific genome-wide DNA methylation changes consistent with Intellectual Developmental Disorder, Autosomal Dominant 7. It is important to note that while a positive episignature result can provide supporting evidence in variant reclassification, a negative episignature result cannot rule out a diagnosis or pathogenicity of a variant (Kerkhof et al., 2024). Furthermore, the confidence of an episignature result and the identification of a corresponding molecular variant should influence the interpretation of episignature findings. A high confidence positive, such as our case, with a corresponding variant is supportive of a diagnosis, however, a moderate confidence positive with a corresponding variant could be a result of a hypomorphic, mosaic, or partially overlapping functional impact variant (Kerkhof et al., 2024). A positive result without a corresponding variant may be the result of an overlapping DNA methylation profile due to a causative variant in a functionally related gene that is not yet clinically defined (Kerkhof et al., 2024). Finally, it should be considered that the strength and reliability of an episignature can depend on the number of reference samples, the diversity of variants within the reference samples, as well as the magnitude of the methylation changes.

The identification of a disease-specific episignature in this patient had immediate and meaningful implications for clinical management and family counseling. A diagnostic confirmation of a *DYRK1A*-related disorder broke the diagnostic odessey by avoiding unnecessary diagnostic assessments and treatment interventions, resulting in savings to the healthcare system. Moreover, establishing a definitive molecular diagnosis allowed the care team to tailor interventions, anticipate potential comorbidities such as seizures or feeding difficulties, and provide more accurate prognostic information to the family. Additionally, knowing the causative variant enabled targeted genetic counseling, informed reproductive planning, and clarified recurrence risks for future offspring. Beyond this individual case, the successful application of episignature analysis highlights its broader potential for other unresolved NDD cases. Integrating episignature profiling into routine diagnostic workflows can accelerate diagnosis, reduce unnecessary interventions and testing, and provide functional evidence to reclassify VUSs. Importantly, episignature analysis does not replace sequence-based testing; rather, it functions as an orthogonal, genome-wide functional assay that can prioritize genes, guide additional testing, and support variant interpretation.

Our findings align with the integrative framework proposed by Courraud *et al.*, which demonstrated that the classification of *DYRK1A* variants requires a combination of clinical, molecular, and functional evidence (Courraud et al., 2021). In their study, variants were classified based on a functional assay demonstrating disruption of autophosphorylation and positive *DYRK1A*-specific DNA methylation pattern (Courraud et al., 2021), which was discovered independently of the *DYRK1A* episignature present on EpiSign. Our case echoes this paradigm, as the patient’s *de novo* exon 5 deletion was initially determined to be a VUS but was reclassified as likely pathogenic through the detection of a strong episignature by EpiSign alongside a fitting clinical phenotype. This emphasizes the value of episignatures analysis as an orthogonal functional assay and highlights its utility in refining *DYRK1A* variant interpretation, preventing misclassification, and improving diagnostic precision.

## 6 Conclusion

This case report provides compelling, real-world evidence for the clinical utility of EpiSign assessment, especially in addressing unresolved cases where a genetic cause remains elusive. For this patient, the episignature assessment served as a critical breakthrough, providing a strong match for a specific syndrome that was consistent with their clinical presentation. This finding reinvigorated our diagnostic search, providing a rationale for further genetic testing by whole-genome sequencing and offering the crucial functional evidence necessary to reclassify the subsequently detected variant of uncertain significance. Without this testing, we would not have pursued further investigation for a variant in *DYRK1A*. Furthermore, subsequent reclassification of the *DYRK1A* variant identified by whole-genome sequencing, testing prompted by EpiSign findings, would likely have been substantially more challenging and prolonged. Ultimately, leveraging EpiSign analysis allowed us to establish a diagnosis, shorten the patient’s diagnostic odyssey, avoid unnecessary testing, and provide the family with a long-sought answer that will guide prognosis, inform long-term care plans, and allow accurate genetic counselling.

## Data Availability

The datasets presented in this article are not readily available because the deposition of individual epigenomic or any other personally identifiable data that has not previously been made publicly available for samples in the EpiSign Knowledge Database (EKD) is prohibited from deposition in publicly accessible databases due to institutional and ethical restrictions. Specifically, these include data and samples submitted from external institutions to the EKD that are subject to institutional material and data transfer agreements, data submitted to London Health Sciences for episignature assessment under Research Services Agreements, and research study cohorts under Institutional Research Ethics Approval (Western University REB 106302 and REB 116108). EpiSign is a commercial software and is not publicly available. Further inquiries can be directed to the corresponding authors or bekim.sadikovic@lhsc.on.ca.
