# Supplementary material for: Episignature leads to diagnosis and reclassification of DYRK1A variant in a child with syndromic neurodevelopmental disorder: a case report
Source: Front Genet. 2026 May 22;17:1813300. doi: 10.3389/fgene.2026.1813300 (PMC13236001; doi:10.3389/fgene.2026.1813300)
Supplement: Supplementary file 1 [file Supplementaryfile1.docx]

**Supplemental Information**

*Additional methods and materials***:** *Whole-Genome Sequencing*

Genomic DNA was enzymatically fragmented and prepared using Illumina-compatible adapter ligation. Libraries were subjected to paired-end sequencing on an Illumina platform, achieving an average genome-wide coverage of approximately 30×. The sequencing reads were aligned with the Genome Reference Consortium Human Build 37 (GRCh37/hg19) and the revised Cambridge Reference Sequence (rCRS; NC_012920) for mitochondrial DNA. The bioinformatics pipeline was performed to detect the variants. This included the DRAGEN platform (Illumina) and tested in-house algorithms to find variants. DRAGEN found single nucleotide variants (SNVs) and small insertions or deletions (indels), while Manta found structural variants. copy number variants (CNVs) were identified by using both DRAGEN-based and in-house methods. Variant prioritization concentrated on rare variants (minor allele frequency <1% in population databases such as gnomAD) and variants previously documented as disease-associated in databases including ClinVar and HGMD. The primary analysis focused on coding regions and adjacent intronic sequences, but the entire gene regions were examined for variants that might be relevant to the clinical phenotype. Interpretation encompassed all possible modes of inheritance and synthesized available clinical and familial history data. Variants were sorted into five groups: pathogenic, likely pathogenic, variant of uncertain significance (VUS), likely benign, and benign. This was done according to established ACMG/AMP guidelines and ClinGen recommendations. Using ACMG/ClinGen standards, CNVs were put into the same categories. Only variants deemed clinically significant to the phenotype were documented. The analytical performance for SNVs and indels showed high sensitivity, specificity, and accuracy (>99.9%) in the areas that were targeted. Detection sensitivity for CNVs was over 95%, but it might not work as well for small events or areas that aren't very complex. When necessary, variants with low sequencing quality or unclear zygosity were put through confirmatory testing using different methods. The ExpansionHunter algorithm was used to look for repeat expansion disorders at known disease-associated loci. A dedicated algorithm was used to look for recombination events in the GBA1 locus, and SMN-specific calling methods were used to look at the number of copies of spinal muscular atrophy (SMA). Uniparental disomy (UPD) screening was performed using an in-house approach based on detection of Mendelian inheritance errors and runs of homozygosity in clinically relevant genomic regions. Overall, ≥99% of targeted genomic regions achieved a minimum coverage depth of 10×, ensuring high confidence in variant detection across the genome.

Genetic findings are interpreted in the context of the provided clinical information, family history, and available laboratory data. Inaccurate or incomplete clinical or genetic information may lead to misinterpretation of results. Only variants in genes relevant to the proband’s reported clinical features are included in the analysis and reporting. Regions with known mapping difficulties in the reference genome, as well as genomic regions that are challenging to sequence with current technologies and lack established relevance to monogenic disease, are excluded from analysis. Complex structural variants, including inversions and translocations, are not assessed by this assay unless explicitly stated in the methods section. Due to inherent technological limitations, certain genomic regions may have low or no coverage. Variants located in repetitive regions, regions of high homology (including pseudogenes), and GC-rich regions may therefore be missed. Low-quality variant calls, including extremely low-coverage calls, are typically considered artefactual based on internal validation studies and are excluded from interpretation. Splice-site and potential splicing-altering variants are evaluated using in silico prediction tools. However, deep intronic variants without strong predicted impact on splicing may not be reported, except in cases where pathogenicity has been established in external databases or literature. Copy number variant (CNV) detection sensitivity is reduced in repetitive or homologous regions and for events affecting two or fewer exons. Mitochondrial variant detection may be limited, and heteroplasmy levels below approximately 15% may not be reliably detected. Repeat expansion analysis is limited to loci with a minimum coverage threshold of 10× and is not designed to resolve complex repeat structures. The Gauchian algorithm detects a defined set of known GBA1 variants and recombination events limited to exons 9–11; variants outside these regions are not assessed. The SMN analysis may not detect all silent carrier states. The uniparental disomy (UPD) detection is performed as a screening approach only and is subject to both false-positive and false-negative results. Finally, a reduced sequencing quality may be affected by sample quality. This includes the Low-quality or compromised samples such as: degraded DNA, hematologic specimens, prenatal samples, and products of conception may prevent certain analyses, including CNV, mitochondrial, or integrated screening assessments.

**Supplemental Information**

*Additional methods and materials:*

*EpiSign Analysis:*

EpiSign uses a support vector machine (SVM)–based classification algorithm that compares the patient’s DNA methylation profile with the EpiSign Knowledge Database (EKD) (14). The EKD includes thousands of clinical, peripheral blood DNA methylation profiles from disorder-specific reference and normal controls (general population samples with various ages and racial backgrounds). The SVM decision values are converted to a methylation variant pathogenicity (MVP) score ranging from 0 to 1, using the Platt scaling method. MVP scores indicate confidence for the specific episignature, with 0 demonstrating no concordance and 1 demonstrating strong concordance. MVP scores greater than 0.01 undergo a secondary review, reflecting the assay’s intentionally sensitive screening threshold designed to minimize false negatives, using Euclidean hierarchical and multidimensional scaling (MDS) clustering plots associated with the episignature. Differentially methylated region analysis of imprinting regions and Fragile X trinucleotide repeat regions assesses methylation changes at the CpG sites present within the regions of interest, compared to unaffected controls and positive controls (meaning those with documented imprinting or Fragile X defects) within the EKD to identify differential DNA methylation.
